# A Review of the Growth of the Fast Food Industry in China and Its Potential Impact on Obesity

**DOI:** 10.3390/ijerph13111112

**Published:** 2016-11-09

**Authors:** Youfa Wang, Liang Wang, Hong Xue, Weidong Qu

**Affiliations:** 1 Systems-Oriented Global Childhood Obesity Intervention Program, Department of Epidemiology and Environmental Health, School of Public Health and Health Professions, University at Buffalo, State University of New York, New York, NY 14214, USA; hongxue@buffalo.edu; 2 Department of Biostatistics and Epidemiology, College of Public Health, East Tennessee State University, Johnson City, TN 37614, USA; 3 Key Laboratory of Public Health Safety, Chinese Ministry of Education, Department of Environmental Health, School of Public Health, Fudan University, Shanghai 200000, China; wdqu@fudan.edu.cn

**Keywords:** fast food, dietary intake, obesity, overweight, policy, China

## Abstract

The fast-food (FF) industry and obesity rates have rapidly increased in China. This study examined the FF industry growth in China, key factors contributing to the growth, and the association between FF consumption (FFC) and obesity. We collected related data from multiple sources and conducted analysis including linear regression analysis on the increase in FF revenue. It was found that FF industry in China is large, with over two million FF facilities. Its total revenue (in million US$) increased from 10,464 in 1999 to 94,218 in 2013, and by 13% annually since 2008. Increased income, urbanization, busier lifestyle, speedy FF service, assurance of food safety, new brands and foods have stimulated demand for FF. Studies have linked FFC with obesity risk, including a few reporting a positive association between FFC and obesity in China. Rapid expansion of Western-style FF restaurants has also stimulated local FF industry growth. Government regulation and public health education need to address the health consequences of rapidly increasing FFC. Lessons learned in China will help other countries.

## 1. Introduction

Rapid economic development, and the growth of global trade and cultural exchanges have accelerated changes in people′s lifestyles in transitional societies like China. Among these changes, the observed shift in people′s food consumption from a traditional diet to a westernized diet is a result of multiple factors, which may contribute to observed increases in obesity and chronic diseases. Over the past two decades, the fast-food (FF) industry and obesity rates have increased rapidly in China [1,2]. Nationwide over one-third of Chinese adults are overweight or obese, while in major cities, like Beijing and Shanghai, more than half are overweight or obese [1].

FF restaurants are also known as quick service restaurants, where customers order items and, in most cases, pay before eating. Food provided by the FF industry includes both Chinese FF and Western FF. Western FF restaurants in China are predominately from the United States (U.S.), such as Kentucky Fried Chicken (KFC), McDonald′s, and Pizza Hut. The number of U.S. FF restaurants has increased remarkably in China. In the U.S., the KFC chain amassed 4618 locations in 61 years, but in China, KFC spreads across 4260 locations in less than 30 years [3]. At present, “Yum! China”, the parent company of KFC, Taco Bell, and Pizza Hut, has approximately 4800 KFCs and 1300 Pizza Huts, with a plan to open 20,000 restaurants in China. McDonald′s is expanding in China at a rate of approximately 10 new restaurants each week. This also indicates how American FF culture has influenced consumers in China [4]. After Western FF restaurants entered China in the mid-1980s, modern Chinese-style FF restaurants have also emerged and developed rapidly as they have learned from the Western advanced management techniques.

Since starting its economic reform in the late 1970s, China′s economy has been growing fast, leading to increased family income, more access to various food choices, faster work pace, and reduced leisure time. The changes have exacerbated the shifts from traditional eating patterns to more modern including Westernized eating patterns featuring high-energy density, high-fat, and low fiber diets [5,6]. The number of people who eat out more frequently due to rapid income growth [7] increased by 40.20% from 2000 (14.70%) to 2008 (20.61%) [8]. Increased Western influences in China include more available FF choices. There has been a shift from traditional full-service restaurants toward FF establishments. Studies have been performed for the association between FFC and obesity. The majority were conducted in Western countries and have demonstrated a positive association [9,10,11,12,13,14]. Similar research is growing in China, but is still limited [7,15,16,17,18,19,20,21,22,23,24].

The rapid expansion of China’s FF industry will likely have many public health consequences, while some have already become alarming as indicated by increasing obesity. FFC may also increase the risk of other chronic diseases such as cardiovascular diseases and diabetes [25]. Compared to the traditional Chinese diet (predominately consists of vegetables and grains, and features plant-based protein, high in fiber, and low in cholesterol and fat), FF often consists of more meats and very limited vegetables, and thus is high in energy density, fat, protein and sodium, and prefers deep frying to boiling [26].

Given China′s large population and the rising obesity prevalence, it is crucial to examine the impact of the FF industry expansion on weight outcomes. However, related studies remain limited and a high level review is warranted. This study examined the increase and patterns of Western- and Chinese-style FF restaurants in China, the factors contributing to the FF industry growth, and the association between FFC and obesity in China. Although they are different, FF access serves as a proxy for FF intake.

## 2. Literature Search

To examine the FF industry growth in China and key factors that might have contributed to the growth, we searched and analyzed data collected from various sources, including government reports, data released by the industry, and published research papers. We searched PubMed (www.ncbi.nlm.nih.gov/pubmed/), Google (www.google.com) and Baidu (www.baidu.com, the largest Chinese search engine). We used key words such as “China”, “fast food industry”, “fast food sale”, and “fast food policy”. In total, 30 papers and reports met our inclusion criteria and were included.

To examine the association between FFC and obesity in China, we searched for studies based on the guidelines of the Preferred Reporting Items for Systematic reviews and Meta-Analyses (PRISMA) [27]. Sources were identified using the PubMed and Medline databases with the following terms: “China”, “fast food, or fast food consumption”, “overweight, obesity, or Body Mass Index (BMI)”. Study inclusion criteria were: (1) publication in English; (2) date of publication between 1990 and June 2015; (3) peer-reviewed sources; (4) use of study subjects who were children, adolescents, or adults; and (5) studies needed to report the association between FFC (or FF access) and obesity (or BMI, waist-to-height ratio (WHtR), waist-to-hip ratio (WHpR)). We also manually searched the reference lists of relevant publications to identify more studies. Information was collected and organized using a standardized data extraction table. The data recorded from each study include reference, region and year of data collection, sample size, sex and age, study design, outcomes (like obesity or/and overweight), and main results (e.g., measures on the association). Among the 39 identified titles, 11 longitudinal and cross-sectional studies conducted in China met inclusion criteria [7,15,16,17,18,19,20,21,22,23,24].

### 2.1. Overview of the FF Industry in China

The modern FF industry in China started when the first KFC restaurant opened in Beijing in 1987. Over the past two decades, the industry grew rapidly, due to rapid developments of China′s economy, urbanization, increased family income, shifts in social norms (e.g., shifting from eating at home to more frequently eating out), strong marketing strategies by the FF industry, increased FF service providers, improvements in chain store and franchising management, and new brands and food choices. The FF industry had estimated revenues of $94.2 billion in 2013, making up about 20.0% of the total catering sub-sector revenue in China.

According to a recent report [3], over two million FF restaurants operated in China in 2013, including franchise and chain operators of all sizes and independent Chinese-style FF facilities. The majority of the enterprises were small and independent facilities that engaged in traditional Chinese-style FF. In recent years, the FF industry revenue grew at an annual rate of about 13.0%.

### 2.2. Major Players and Market Composition of FF Restaurants in China

The majority of FF restaurants in China are privately owned (e.g., 76% in 2012). American FF restaurants expanded rapidly in China, especially in the past decade. The following key Western FF restaurants show the expansion [28]: (a) KFC is the largest foreign brand in China in terms of the number of restaurants, which were distributed in over 1000 cities in 2015, covering all provinces in China except for Tibet; (b) McDonald′s opened its first restaurant in Shenzhen in 1990. In 2013, it owned over 2000 outlets; and (c) Pizza Hut opened its first store in Beijing in 1990. In 2015, Pizza Hut became the largest Western casual dining brand in China.

Domestic Chinese style FF restaurants in China have expanded as well, and have been largely affected by Western FF restaurants. They together have stimulated demand for FF in China [29]. The main types of Chinese style FF restaurants include: (a) the Malan Noodle FF Chain, founded in 1995, is the largest domestic FF chain, but is much smaller than the American FF chains like KFC and McDonald′s. In 2008, Malan Noodle had over 400 chain stores in China [28]; (b) Chinese-style rice sets, quick-service restaurants and take-out facilities operate independently and are located in various settings including shopping centers and streets. They account for over one-third of the FF industry revenue [28]; and (c) there are other Chinese-style noodle and dumpling stores that are particularly preferred for breakfast and lunch. These key domestic FF restaurants generated 43% of the total FF industry revenue in 2013 [28].

### 2.3. Growth of China’s FF Industry

**Figure 1:**
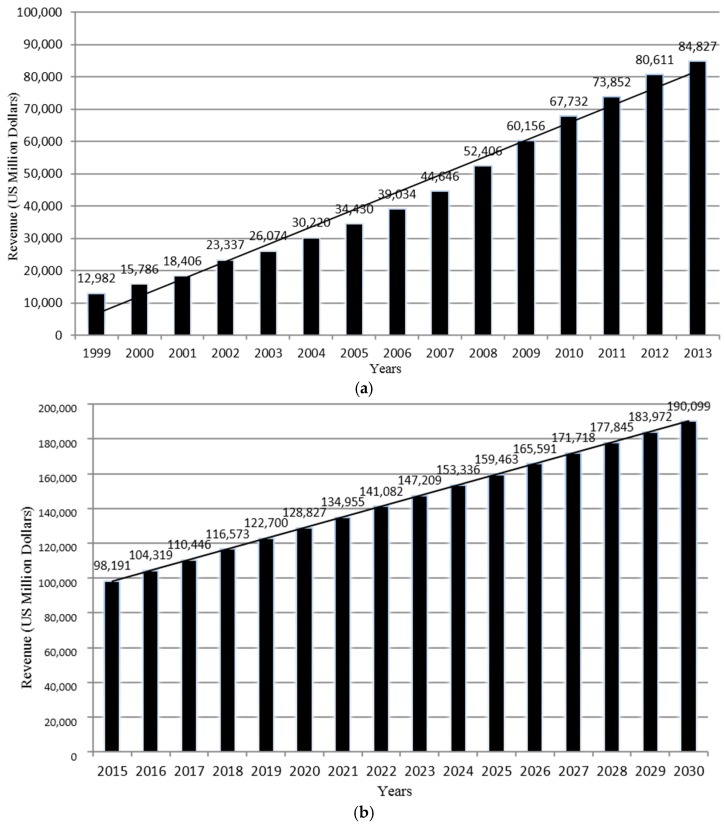
Trends in fast food (FF) industry revenue growth and future projection in China between 1999 and 2030. (**a**) Observed FF industry revenue growth in China between 1999–2013 adjusted for the inflated by Consumer Price Index, 2010 = 100); (**b**) Projected FF industry revenue growth in China between 2015–2030.

China’s FF industry has been growing rapidly, especially over the last decade (see Figure 1 and Table 1). Many American FF enterprises have entered and expanded in the Chinese market since the 1990s. This has promoted new concepts of chain restaurant management and franchising, food supply management, and other related FF techniques. FF restaurants with strong brand-name images, such as KFC and McDonald′s, are more popular than local Chinese-style FF, because they are known for standardized quality control and effective store management. Western-style FF outlets are particularly favored among adolescents and young adults [28].

Foreign enterprises have intensified the FF industry competition and posed challenges for local restaurants. Meanwhile, they have brought new operating models and consumer conceptions, stimulating FF industry development. The success of Western FF restaurants will continue to serve as a catalyst for the future development of the FF industry in China, in terms of the number and types of restaurants.

Lifestyle changes, including faster pace of work and life, have prompted demand for FF. Other factors may include the rapid development of FF service providers, improvements in chain store and franchising management, taste preference or low cost, and new brands and food products (e.g., KFC creates new food products only in China such as Old Peking Children Wrap to favor the Chinese market).

The rapidly growing industry has caused intensive competition. McDonald’s and Dico′s open a new restaurant every three days. Outlets of domestic and Asian FF operators, such as Malan Noodle, Yonghe King, New Asia, and Yoshinoya, have been increasing with a slightly slower rate. Enterprises in the industry have grown both in numbers and scale (e.g., revenue). The number of FF restaurants in the Top 100 catering enterprises increased from 20 in 2004 to an estimated 30 in 2010, while their share of revenue increased from 33.0% to 36.0% [28].

#### Future Growth of China′s FF Industry

Growth potential remains high for the years to come thanks to the high and growing demand for FF and profitable pricing. It is projected that between 2008 and 2018 the IVA would increase at an annual rate of 10.7%, while China′s GDP annual growth rate is 8.0% and may slow down [3]. Based on reported FF data, we projected future FF revenues for 2015–2030, assuming the trend would continue in the future (Figure 1). We fitted linear regression model using time as the predictor and the model fit well (R^2^ = 95%). ACMR-IBIS World, the largest provider of industry information in the U.S., also forecasted that in the next five years, industry revenue would increase by 8.9% annually to reach $144.3 billion in 2018 [3]. Likely the FF chain operations will expand in less-developed regions, and continue spreading from the East coast to the West inland regions, and from large to small cities and towns.

### 2.4. Key Factors That Contributed to the Growth of FF Industry in China

A set of factors may have contributed to the rapid growth of the FF industry.

#### 2.4.1. Key Demand Drivers

##### Economic Development

Economic development has changed people′s lifestyle, fueling the FF industry growth. The geographic spread of the FF industry parallels with China′s economic development, in particular in major cities in China.

##### Large Population Size

China is the largest FF market in the world due to its large population size. The process of urbanization further contributes to the industry development, particularly for modern enterprises using advanced technology, which may lead to less healthy diets.

##### Increased Household Income

Increased household income may increase food consumption, especially eating out, stimulating the industry growth. Fast economic growth due to economic reform resulted in a fast increase in income, which has fueled dramatic shifts in food behaviors and dietary patterns. The per capita disposable income increased by 11 times after accounting for inflation between 1978 and 2012, which increased from about 340 RMB to 24,500 RMB. The income increase has related to a significant increase in the consumption of food-away-from-home (FAFH) including FFC [30,31]. In the mid-1990s, urban household average per capita annual FAFH expenditure was <200 RMB, which has increased to about 1000 RMB in 2010 [32].

##### Lifestyle Changes Due to Economic Development, Urbanization, and Influence of Western Culture

The fast economic development and rapid urbanization have resulted in faster lifestyles; and thus FF has become increasingly popular. Chinese consumers, especially those who live in large cities, more easily embrace Western-style FF restaurants. In provinces and regions of better economic development and faster lifestyles, FF restaurants account for a much larger share of the total food-service sector′s revenue. For instance, in Guangdong (a rich province in south China), the FF market accounts for about 90%, while in large cities such as Beijing, Shanghai and Tianjin, the share is over 50%.

##### Increased Leisure and Recreational Activities and Time

Increased leisure time and fast daily life pace stimulated the demand for FF. Many people enjoy eating out with their families or friends and often combine eating out (including eating in a FF restaurant) with other activities such as shopping and seeing movies.

##### Popularity and Peer Influence

The high popularity and acceptance of FF among young people is a strong contributor for demand for FF, especially Western FF. Western FF emphasizes an exciting dining atmosphere and fast service, which is different than traditional Chinese food that focuses more on taste, tradition and content, but pays less attention to the eating environment. Hanging out in Western FF restaurants is favored by youth and young adults, as well as in low- and middle-income groups in China [33], which promotes a pro-FF social environment, especially among young people.

##### Health Consciousness and Concerns of Food Safety

Increasing health consciousness can increase the demand for FF. Food products served in Western FF restaurants are often perceived as safer than those served in small, local restaurants. However, in recent years, more Chinese have become aware of some undesirable respects of FF such as that FFC may increase obesity risk. This may reduce demand for FF.

#### 2.4.2. Key Supply Drivers

##### Reforms in China′s Agricultural and Trade Sectors Have Facilitated Rapid FF Industry Growth

Three major reforms have been conducted in the agricultural sector in China: (1) the People′s Commune System was reformed and replaced by the Household Responsibility System (in early 1980s); (2) marketing systems reforms for agricultural commodities and input factors (starting from the late 1980s); and (3) rural taxation system reform (starting from 2000). The agriculture tax was cancelled in 2005 and since summer 2007, the government has started to subsidize the production of hogs, dairy cattle, and edible oils. China joined the World Trade Organization (WTO) in December 2001. As part of its WTO commitments, China was expected to reduce overall average agricultural tariffs from 22% to 17% by 2004. China agreed to reduce tariffs on U.S. priority agriculture products from 31% to 14% by 2005. For example, the tariff on beef should be reduced from 45% to 12%, and poultry from 20% to 10% [34]. These policy changes have led to the liberalization of domestic markets and international trade, narrowed the domestic-international price gaps, and stimulated the domestic production and promoted imports, which facilitated the vertical integration of the FF industry and helped largely reduce FF ingredient costs. In addition, China′s transformation from a rural agriculture economy to an industrialized economy has provided the FF industry with vast cheap labor supply to help it to expand.

##### China′s FF Industry Regulations Are Loose

Barriers to entering this industry are generally low, although they are increasing due to the higher technology requirements for chain operators. Progress in the legal framework applicable to the FF industry was made in 2005, when the Ministry of Commerce issued the new Measures for the Administration of Commercial Franchises. This new regulation abolished the legal uncertainty regarding franchising in China, and encourages foreign franchisors in the retail, sport, restaurant, hotel and other service industries with business tax exemptions and other preferential tax conditions [3]. The legal framework improvement has had a positive influence on the FF industry development and encouraged new market entries, as the franchise operation model lowers the entry barriers for both franchisors and franchisees. In part due to the legal changes regarding franchises, foreign FF enterprises have grown faster than the industry as a whole over the past decade. With their strong management, technological expertise and capital strength, foreign enterprises are better positioned than their domestic competitors.

##### Western FF Chains and Operation Stimulated Local FF Industry Growth

The increasing influence of foreign enterprises has enhanced the domestic FF industry development and modernization by introducing new operation models and food concepts. Local companies become more competitive after they follow Western FF examples by trying to establish their own brands and networks. Additionally, they have the advantage of offering traditional Chinese food, which has higher nutritional value and lower prices. Now that local companies have been operating in the market for many years, there are fewer failures and more successful operators.

##### The FF Industry, Especially Western FF Chains, Has Made Vigorous Marketing Efforts

Western FF companies, for example, KFC and McDonald′s, have been promoting FFC to young people aggressively as a modern and elite-class lifestyle through advertising. The FF restaurants are depicted as romantic and happy places for young people to eat in and be social. Television commercials are directly targeted at children without any age restrictions. Coupon incentives have been intensively used to increase sales. Due to increasing and intensive FF advertisements, Chinese children are devouring the American FF. In addition, the Western-style FF companies also took strategies to adapt to local food culture and practice. Localization of their menus is a key factor that has promoted the growth of Western FF in competing with local competitors. For example, McDonald′s in 2012 started to sell soymilk which is a popular traditional Chinese breakfast beverage. KFC has spicy chicken products in the Sichuan region where local people enjoy spicy food.

### 2.5. Increase of Obesity Prevalence in China

**Figure 2:**
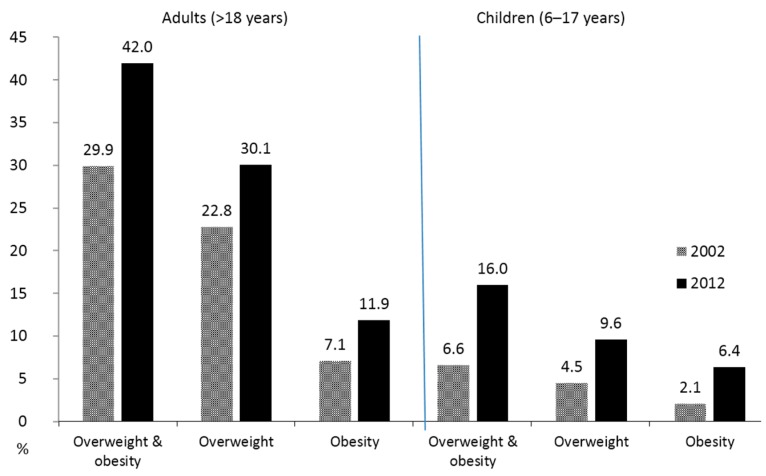
Increase of prevalence (%) of overweight and obesity in China among adults and children from 2002 to 2012. Based on national representative data and Chinese BMI cut points, e.g., for adults, 24 < BMI < 28 for overweight, and BMI>28 for obesity; for children, age-sex-specific BMI cut points were used.

Over the past two decades, obesity has been increasing rapidly in China. National data show that the prevalence of overweight and obesity among adults increased from 20% in 1992 to 30% in 2002 and 42% in 2012 based on the Chinese BMI standard (Figure 2) [1,35,36]. This increase paralleled that of FFC.

### 2.6. Association between FFC or Access to FF and Obesity or Related Outcomes

Some studies have examined the association between FFC or access to FF and obesity (or obesity-related measures like BMI, WHtR, WHpR) in China. Seven were based on regional data and nine were cross-sectional studies, while only two were longitudinal studies (see Table 2). Overall, the studies indicate a positive association between FFC and obesity. Our previous study of over 24,000 children aged 2–18 in Beijing found that children consuming western FF ≥ 3 times per week were 1.50 times (95% CI = 1.12–2.02) as likely to be overweight or obese compared to children with FFC < 1 time per week [17]. Among female adolescents in Xi′an City, having breakfast outside the home (often likely consuming local FF) was positively associated with overweight and obesity (OR = 1.7, 95% CI = 1.1–2.3) [15]. Among Hong Kong Chinese adults, eating out at least twice a week as compared to <2 times a week was positively associated with being obese (OR = 1.25, 95% CI = 1.06–1.49) [16]. A notable longitudinal study (during 2000–2009) examined the associations between changes in BMI, WHtR and WHpR and changes in Western FF restaurants in 216 communities for more than 9000 Chinese adults from nine provinces in China [19]. Results revealed positive associations between the number of Western FF restaurants and subsequent increases in central adiposity in both urban and rural populations, but did not detect a positive association with BMI. Future studies in China using national and longitudinal data are warranted to examine the influence of FFC on obesity risk.

Food products served in FF restaurants in China, especially Western style ones, usually have high saturated fat, high energy density, glycemic loads, simple carbohydrates, sugar, and large portion sizes. In contrast, a traditional rural Chinese diet features plant-based protein, low cholesterol, and some dietary fat [26].

## 3. Discussion

China′s FF industry is large and is expanding rapidly, which parallels the rapid increase in obesity, urbanization and economic development. Over two million FF facilities operated throughout China in 2014. The FF industry made up about 20% of China′s total catering sub-sector revenue. The industry has been growing at a remarkable rate over the past three decades, especially over the past decade and in urban areas. At present, FF facilities remain predominately in urban areas. The number of FF stores in rural areas is still small, but will likely increase quickly in the future. Multiple factors have stimulated the demand for FF such as increased family income, urbanization, busier lifestyle, FF speedy service, concerns of food safety, new brands and foods, FF industry′s vigorous and effective marketing, and changing social norms and values. Overall, the Chinese central and local governments′ regulations on FF industry are light. Some local governments have encouraged its growth, in particular, due to their desire to increase economic growth and tax revenues. Some groups such as those living in urban areas, having higher income, being adolescents, and being boys are more likely to consume FF.

Studies examining the association between FFC and obesity are predominately conducted in Western countries and many have reported a positive association between the number of FF restaurants and FFC and obesity [9,10,11,12,13,14]. A systemic review based on 16 studies conducted in Western countries examined the association between FFC and risks of weight gain and obesity, and revealed mixed results [11]: six of the seven prospective cohort studies and four of the six cross-sectional studies showed a positive association between FFC and energy intake or BMI; among the three experimental studies, a randomized intervention trial among 891 U.S. women showed that an increase of one FF meal per week was associated with weight gain of 0.72 kg over the 3-year period (*p* = 0.01).

The other two studies (i.e., a feeding trial and a subsequent crossover study) conducted among adolescents did not find a significant association. A recent study reported the impact of FFC on mean population BMI and the possible influence of market deregulation on FF and BMI based on data collected from 25 high-income countries [12]. This study suggests market deregulation policies may contribute to the obesity epidemic by facilitating the spread of FFC. Another U.K. study reported a positive association between FFC and obesity (OR = 1.23, 95% CI = 1.02–1.49) among 4837 children aged 13–15 [10]. Obesity prevalence increased with frequent eating in FF restaurants among a U.S. study sample, from 24% of those who ate in FF restaurants less than once a week to 33% of those who did it ≥3 times per week [9]. Similar research is growing in China [7,15,16,17,18,19,20,21,22,23,24], showing the fast expansions of FF industry and increased FFC likely have contributed to the increase of obesity in China. Overall, increasing studies in Western countries and China have reported a positive association between FFC and obesity [7,9,10,11,12,13,14,15,16,17,18,19,20,21,22,23,24].

However, longitudinal studies regarding the relationship between FF and weight status is still largely lacking in the literature. We noted that some studies in China have examined the influence of FFC on obesity. Most of them reported a positive association, but are predominately cross-sectional studies based on regional data [15,16,17,18,19,20,21,22,23,24]. Eight of these 11 studies only used regional data [15,16,17,18,21,22,23,24]. The other three studies [7,19,20] used data from the China Health and Nutrition Survey (CHNS), which is not a nationally representative study. Other methodological issues of some of these CHNS-based studies are noticed. For example, despite experiencing transitions of retailing environment among the surveyed regions, the progressive nature of the most rapidly changing regions cannot be fully captured where the prevalence of FFC is higher. The food retailing environment just used the number of food operating business as an indicator, but did not take account into the type of food or operation [20]. Future studies need to provide details for the changing food environments.

Other behaviors may result in weight gain due to overeating or reduced physical activity, which are associated with obesity, including eating occasions away from home, large portion sizes, high consumption of beverages high in sugar, and not having breakfast [37,38,39,40,41,42,43]. In addition to these behavioral factors, multistructural variables (e.g., the physical environment and SES) have been shown to play a role in food intake and energy expenditure [44,45]. Environmental influences, e.g., large supermarkets provide healthy foods at reasonable prices, may influence food purchasing behaviors [46].

In China, the increase of FFC is being fueled by the substantial increase in supply. The fast expansion of the FF industry has made it much easier for consumers to access FF at relatively lower costs, including lower price, time saved, and travel convenience. Despite the potential adverse effects of FFC on health, there is lack of policies and regulations in China to monitor and regulate the FF industry. There are many unregulated or illegal operators besides the 1.35 million enterprises registered by the China Commercial Association and China Cuisine Association. Over the past three decades, rapid economic development has dramatically altered the food landscape in China. The adverse health effects associated with economic and FF industry growth is a result of the negative externality induced by market reform related to food supply and demand. This necessitates a better understanding of the FF industry structure and food demand system and related health consequences in China.

During recent years, China passed several laws and regulations related to the FF industry. China′s Food Safety Law was passed on 28 February 2009, and has taken effect since 1 June 2009. This law is formulated to ensure food security and prescribes safety standards of food, regulations on food production, food examination, food imports and exports, and disposal of contaminated food. In 2011, “Food Safety Standards of Fast-food Service” was issued to regulate the employment, selection of store location, management personnel, equipment preparation, and process control of FF stores. The laws and regulations concerning franchise businesses were initially established in November 1997, when the Ministry of Internal Trade published the first Chinese franchise law—the Regulation on Commercial Franchise Business. This was initially a trial implementation and included important legal issues such as trademarks, copyrights and intellectual property protection. Since then, the regulation has been modified and enhanced. The latest version of the franchise rules, Measures for the Administration of Commercial Franchises, was issued by China’s Ministry of Commerce and became effective on 1 February 2005. This new regulation replaced the first franchise law and became the only legal framework for franchising in China. However, these laws and regulations were not driven by public health goals.

Studies have observed the outcomes of laws and policies enacted in other countries to help reduce FFC or encourage FF restaurants to sell healthier foods. One study conducted a microsimulation analysis on the federal policy in the U.S. that banned child-directed FF television advertising [47]. The microsimulation determined that the ban would reduce childhood obesity by almost 1% [47]. While this is the lowest percentage found, they discovered that it would have the greatest predicted behavioral impact [47]. Another study looked at cardiovascular disease prevention, including improved diet, through policies. They found that mass media campaigns and limiting marketing towards children can improve diet [48]. They also discovered that comprehensive worksite wellness interventions can improve diet [48]. Therefore, workplace policies as well as advertising policies could potentially reduce FFC or lead to the selection of healthier menu items. One large policy enacted required restaurants to label their menus with the energy contents of the food. A systematic review observed the effects of menu labelling by SES [49]. The authors determined that this policy significantly reduced the number of calories purchased from people in the high SES neighborhoods as well as those visiting higher SES neighborhoods [49]. However, more studies need to be conducted to determine the effect on multiple SES neighborhoods. Enacting similar policies and laws in China could contribute to reducing FFC and obesity.

More public health perspectives in terms of the prevention of non-communicable chronic diseases should be incorporated into China′s FF industry regulations. Nutrition standards based on the Dietary Guidelines for Chinese Residents should be implemented to require the FF industry to provide healthier food choices. Standardized food labeling with easily readable and understandable content and format should be enforced in FF restaurants to assist consumers in making informed decisions.

This study has some limitations. FF restaurants and revenue indicate FF access, but FF access and FF consumption are different. Some questions remain unclear such as: how has FF intake increased in China? What percent of daily calorie intake is provided by FF among Chinese people? How do various population groups in China consume FF? What are the top food and beverage items purchased and consumed from FF restaurants? Future studies regarding portion sizes, energy density, fat, or added sugar content of FF consumed by Chinese children or adults are needed.

Future research is needed to help answer the above questions and to help inform policy makers to develop cost-effective fiscal and public health policies taking into account the complexity of the demand and supply actors. For example, taxing FF and subsidizing healthy food consumption is paid increased attention in developed countries. Chinese central and local governments need to consider such options. Also, FF advertising should be well regulated such as limiting its reaching out to children and other vulnerable populations. The establishment, implementation, effectiveness, and sustainability of these policies and regulations crucially depend on the awareness and perception of the risk of FF on health of related stakeholders and the whole society. How to balance economic returns and future public health costs is a challenge faced by the Chinese central and local governments.

## 4. Conclusions

The rapid growth of the FF industry and FFC in China has become a public health concerns considering the negative health consequences of FFC, including related obesity risks. Future research needs to examine the impact of the FF industry growth and of people’s FFC on health, and to assess the influence of related existing and new government regulations on the growth and operation of the FF industry. Other growing economies are likely to face the similar challenges as China does. The lessons learned in China will help other countries.

## Figures and Tables

**Table 1:** Key statistics and growth of the fast food industry in China: observed for 2004–2012.

Year	Revenue ($m)	IVA ($m)	Establishments (Units)	Enterprises (Units)	Employment (Units)	Wages ($m)	Assets ($m)
2004	25,694.2	6475.0	929,125	925,946	2,651,296	3376.9	23,638.7
2005	29,804.7	7719.3	1,128,886	1,124,984	3,370,846	4384.2	27,420.3
2006	34,285.5	9016.9	1,358,050	1,353,467	4,207,130	5516.5	31,199.9
2007	41,084.6	10,355.0	1,624,228	1,618,876	5,024,730	6886.0	36,565.3
2008	51,070.9	12,357.1	1,761,543	1,752,945	5,643,072	8217.3	41,878.1
2009	58,220.9	13,839.9	1,814,736	1,800,367	6,015,178	9376.3	45,647.1
2010	67,732.0	16,238.6	1,896,399	1,872,382	6,466,316	10,752.1	50,211.9
2011	77,891.8	18,755.5	1,958,900	1,927,656	6,886,627	12,082.4	54,982.0
2012	87,238.8	21,100.0	2,015,856	1,981,019	7,299,824	13,397.1	59,380.6

Data source: Fast-Food Restaurants in China. (2014) China Fast-Food Restaurants Market: New market research published [3]. Abbreviation: IVA, industry value added, or the value added of an industry, also referred to as gross domestic product (GDP)-by-industry, is the contribution of a private industry or government sector to overall GDP. The components of value added consist of compensation of employees, taxes on production and imports less subsidies, and gross operating surplus. Value added equals the difference between an industry′s gross output (consisting of sales or receipts and other operating income, commodity taxes, and inventory change) and the cost of its intermediate inputs (including energy, raw materials, semi-finished goods, and services that are purchased from all sources).

**Table 2:** Key study characteristics and findings regarding the association between fast food consumption (FFC) and obesity in China.

References	Region; Year of Data Collection	Sample Size, Sex, and Age	Study Design	Outcome (Prevalence)	Main Results such as Adjusted OR (95% CI) for the Association between FFC and Overweight/Obesity
[7]	Nine provinces in China, the China Health and Nutrition Survey (CHNS); data were collected in 2004, 2006, 2009, and 2011.	24,396 adults (11,835 men and 12,561 women); 18 years or older	Longitudinal study	BMI	Among men, an increase of one indoor restaurant in the neighborhood was associated with a 0.01 unit increase in BMI, and an increase of one fixed outdoor food stall was associated with a 0.01 unit decrease in BMI. Among women, an increase of one indoor restaurant in the neighborhood was associated with a 0.005 unit increase in BMI, and an increase of one FF restaurant and one fixed outdoor food stall was associated with a 0.02 unit and 0.004 unit decline in BMI, respectively.
[19]	Nine provinces in China, CHNS; data were collected in 2000, 2004, 2006, and 2009.	29,116 adults (13,993 men and 15,123 women); 18 years or older	Longitudinal study	BMI, waist-to-height ratio (WHtR) and waist-to-hip ratio (WHpR)	Number of Western FF restaurants was positively associated with subsequent increases in WHtR and WHpR among rural population. More FF restaurants were positively associated with a future increase in WHpR for urban women. Increased availability of FF between two waves was associated with increased WHtR for urban men over the same period. A past increase in number of FF restaurants was associated with subsequent increases in WHtR and WHpR for rural population.
[21]	Kunming, China; Data were collected in 2011.	575 adolescents (sex specific sample size was not provided); 13 years or older	Cross-sectional study	BMI, overweight, obesity	Proximity to FF was positively associated with higher BMI. Adolescents who lived in the more developed inner neighborhoods had a higher prevalence of overweight.
[20]	Nine provinces in China, CHNS; data were collected in 2006.	9788 adults (4659 men, and 5129 women); 18 years or older	Cross-sectional study	Overweight/obesity	The relationship between FFC and overweight/obesity was irrelevant for Chinese segments that did not have access to FF. Factors that were most associated with segments with a higher BMI were consumers′ (incorrect) dietary knowledge, the food retail environment and sociodemographics.
[18]	Five primary and middle schools, Tianjin, China; data were collected in 2010.	3140 school children and adolescents (1559 boys and 1581 girls); 7–18 years	Cross-sectional study	Overweight/obesity	Having lunch in FF restaurant (versus home) was positively associated with overweight (OR = 2.03, 95% CI = 1.34–3.07).
[22]	The Elementary School Children’s Nutrition and Health Survey; Taiwan, China; data were collected in 2011–2002.	2283 school children (1189 boys and 1094 girls); 6–13 years	Cross-sectional study	General obesity (based on BMI) and abdominal obesity (based on waist circumference)	A high FF stores density was associated with higher BMI and abdominal obesity in boys, but not in girls.
[17]	The Child and Adolescent Metabolic Syndromes Study (BCAMS), Beijing, China; data were collected in 2004.	21,198 children (10,602 boys and 10,596 girls); 2–18 years	Cross-sectional study	Overweight/obesity	Children with western FFC ≥ 3 times per week were 1.50 times (95% CI = 1.12–2.02) as likely to be obese compared to children with FFC < 1 time per week.
[15]	Xi‘an City, China; data were collected in 2004.	1792 adolescents (899 boys and 893 girls); 11–17 years	Cross-sectional study	Overweight/obesity	Having breakfast outside the home (often likely consuming FF) was associated with overweight and obesity among females only (OR = 1.7, 95% CI = 1.1–2.3).
[23]	Xi’an, China; data were collected in 2004.	1792 adolescents (899 boys and 893 girls); 11–17 years	Cross-sectional study	Overweight and obesity	The odds of overweight and obesity was 1.8 times (95% CI = 1.1–2.9) greater if the parents decided to have Western FF than if the children decided by themselves.
[24]	Changsha, China; data were collected in 2007.	4140 students (2209 boys and 1931 girls); 7–12 years	Cross-sectional study	Obesity	Increased consumption of fried foods was associated with obesity.
[16]	The “Better Health for Better Hong Kong” (BHBHK) Campaign, Hong Kong, China; data were collected between July 2000 and March 2002.	4841 adults (2375 men and 2466 women); 17–83 years	Cross-sectional study	Obesity	Eating out at least twice a week as compared to less than 2 times a week was associated increased odds of obesity (OR = 1.25, 95% CI = 1.06–1.49).
